# Epidemiological characteristics and spatial-temporal clusters of hand, foot, and mouth disease in Qingdao City, China, 2013-2018

**DOI:** 10.1371/journal.pone.0233914

**Published:** 2020-06-05

**Authors:** Zengqiang Kou, Jing Jia, Xiaohui Liu, Tingting Luo, Xueling Xin, Jinling Gong, Jingfei Zhang, Dapeng Sun, Fachun Jiang, Ruqin Gao

**Affiliations:** 1 Shandong Center for Disease Control and Prevention, Jinan, China; 2 Department of Acute Infectious Disease, Qingdao Centre for Disease Control and Prevention, Qingdao Institute of Prevention Medicine, Qingdao, Shandong, China; 3 Department of Public Health, Qingdao University Medical College, Qingdao, Shandong, China; Faculty of Science, Ain Shams University (ASU), EGYPT

## Abstract

**Background:**

Hand, foot, and mouth disease (HFMD) has become one of the most important infectious diseases recent years. Qingdao City has suffered from serious HFMD epidemic. This study aimed to describe epidemiological characteristics and investigate spatial-temporal distribution at town level in Qingdao City.

**Method:**

The surveillance data of HFMD during 2013–2018 were collected from the National Notifiable Disease Surveillance System. The global Moran’s *I* statistic was used to detect the spatial autocorrelation of HFMD cases by ArcGis 10.0 software. Purely spatial and spatial-temporal analysis was used to detect epidemic clusters by SatScan^TM^ v9.6 software.

**Results:**

The annual average incidence of HFMD cases in Qingdao City from 2013 to 2018 was 123.16 per 100000, while the incidence rate of children≤5years old was 2879.80 per 100000. The majority (88.97%) of HFMD cases were aged within 0–5 years old and the males were 60.20%. Other enterovirus (EV), enteriovirus 71(EV71), and Coxsackievirus A16 (CA16) accounted for 48.75%, 30.91% and 20.34%. The seasonal peak was between May and October. HFMD had positive spatial autocorrelation at town level with global Moran’s *I* from 0.19 to 0.31(P<0.001). Spatial-temporal cluster analysis detected six most likely clusters and three secondary clusters from 2013 to 2018. The most likely cluster was located in urban and urban-rural fringe areas.

**Conclusions:**

Urban and urban-rural fringe areas were the major locations of the clusters with other EV as the dominant pathogen between May and October. The findings suggested that the prevention and control of HFMD in Qingdao City should be focus on these high-risk periods and locations which had important public health significance for the allocation of public health resources.

## Introduction

Hand, foot, and mouth disease (HFMD) is a common intestinal infectious disease caused by viruses that belong to the Enterovirus group principally including Enterovirus 71(EV71) and Coxsackievirus A16 (CA16) [1, 2]. The clinical symptoms of HFMD are characterized by fever, oral ulcer, vesicular exanthema on hands, feet, and mouths. The transmission of HFMD is mainly from person to person through direct contact with saliva, respiratory droplets, faeces, vesicular fluid of patient and indirectly by contaminated articles [3]. Symptomatic cases mostly affect children aged 0–5 years, but also can affect older children and adults. Most cases are mild and self-limiting, but few cases may develop severe complications involving neurological symptoms such as encephalitis, meningitis and even death [4, 5].

In the last decade, there are a large number of HFMD outbreaks reported in East and Southeast Asia, including China [6–8]. Large-scale HFMD outbreaks were reported in Shandong and Anhui Province in 2007 and 2008, which resulted in a large number of server cases and deaths [9, 10]. Many spatial analysis had been carried out to describe spatial patterns and cluster locations in many provinces in China, such as Shandong, Beijing, Sichuan, and so on. These studies mainly focused on the analysis of the epidemiology characteristics, global or local spatial autocorrelations and spatiotemporal clusters. In order to better deal with the outbreaks of HFMD, the Ministry of Health of China listed HFMD as a Class C statutory infectious disease on May 2, 2008 [11, 12]. In addition, the inactivated monovalent EV71 vaccines were licensed in China in 2016 [13], which provide effective measures to prevent HFMD caused by EV71, especially severe cases.

The HFMD epidemic in Qingdao City exhibited an upward tendency with the incidence rates ranged from 31 in 2008 to 138 in 2018 per million person-years, which was much higher than that of Shandong Province with the average rate of 10.44 per million person-years (1.87~32.84 per million person) during 2008 to 2012. However, previous studies about HFMD in Qingdao City mainly concentrated on descriptive studies and forecasting analysis [14, 15]. There are no clear analysis on the spatiotemporal characteristic at town level, which is not conductive to take targeted preventive and control measures. Thus, based on the data from the surveillance system, we performed the scan statistics analysis to explore the town-level epidemiological characteristics and spatial-temporal distribution of HFMD in Qingdao City, which could provide reference for the accurate prevention and control of HFMD and guide the optimal allocation of social health resources.

## Material and methods

### Study area

Qingdao City is one of the largest tourist port cities in China with a population of 9.20 million people. The city covers an area of 11282 km^2^, which is divided into 10 districts including 130 streets/townships. According to the geographical characteristics, the 10 districts were divided into three regions including urban areas (Shinan District, Shibei District and Licang District), urban-rural fringe areas (Chengyang District, Laoshan District, Jimo District, and Huangdao Distirct) and rural areas (Pingdu District, Laixi District and Jiaozhou District).

### Data collection

The surveillance data of HFMD from 2013 to 2018 were obtained from the National Notifiable Disease Surveillance System. The information included gender, age, occupation, address, onset date, diagnosis date and the pathogen type (CoxA16, EV71 and other EV). The clinical criteria for diagnosis of HFMD cases was provided in the HFMD Control and Prevention Guide published by the Chinese Ministry of Health [11].The range of time was from January 1, 2013 to December 31, 2018. The cases were diagnosed and reported to the surveillance system by professional doctors. Throat swabs were collected from at least 5 cases in each district. All swab samples were stored at 4°C immediately after collection and quickly sent to the national network laboratory in Qingdao for aetiological indentification. EV71, CA16, and other EV were tested by real-time PCR. In this study, we focused on the HFMD cases (0 to 5 years) which accounted for about 90% of the total cases. The corresponding demographic data of each town were obtained from Qingdao Statistical Yearbook.

### Statistical analysis

Descriptive statistics were used to describe the epidemiological characteristics of HFMD. Demographic characteristics of HFMD cases and the pathogen types of some cases were analyzed by year and seasonal pattern by month. The town-level polygon map at 1:100000 scale was obtained to perform the spatial distribution by ArcGIS 10.0 (http://www.arcgis.com).

The autocorrelation statistic (Moran’s *I*) [16] was used to detect the global spatial autocorrelation of HFMD cases in the study area by year. ArcGIS 10.0 was used to compute Moran’s *I* test statistic [17,18].

The spatial and space-time scan statistics were used to identify high-incidence clusters of HFMD in Qingdao City during 2013 to 2018 [19]. The analysis was performed by SatScan^TM^ v9.6 software (http://www.satscan.org/), using the kulldorff method of retrospective space-time scan statistic based on a discrete Possion model [20]. Spatial scan statistic was used to identify purely spatial clusters of HFMD cases. The purely spatial scan statistic applied a circular window that centered on each geographical area. According to the population range (specified by user), the radius of the window varied continuously in size. The space-time scan statistic was defined by a cylindrical window with a circular geographic base and with height corresponding to time [21, 22]. The base and the height of the windows are in dynamic changes in order to detect possible spatial-temporal clusters. The scan statistic was based on the null hypothesis that the rate or the independence of cases in space and time was the same within and outside the scanning window. The Log Likelihood Ratio(LLR) and relative risk(RR) were calculated to test the hypothesis. The P value(P<0.05) was obtained with Monte Carlo hypothesis testing with 999 simulations [23]. The window with the maximum LLR was considered as most likely cluster, the others were defined as secondary clusters.

For this study, we analyzed 130 towns of Qingdao City in 72 months from January 2013 to December 2018. In order to find possible clusters, the maximum radius and height were all set to 50% of the total population at risk in the total study period. The results of scan statistic analysis were visualized by ArcGis 10.0.

## Results

### Demographic characteristics

A total of 69646 HFMD cases were reported in Qingdao City from 2013 to 2018 including 1635 severe cases and no fatal cases. The average annual incidence rate was 123.16 (ranged from 68.05 in 2013 to 146.90 in 2015) per 100000 inhabitants.

During the study period, the number of children ≤5 years old accounted for the largest proportion (from 86.50% to 90.67%) among these cases, with the annual average incidence rate 2879.80 (ranged from 1725.45 in 2013 to 3667.06 in 2014) per 100000 inhabitants (Table 1). Thus, we mainly conducted the following spatial analysis on children≤5 years old.

**Table 1:** Demographic characteristic of HFMD cases and the pathogen types of some cases in Qingdao City, 2013–2018.

	2013	2014	2015	2016	2017	2018	Total
Age							
0–5 year	5606	11224	12448	10116	11048	11471	61913
>5 year	636	1199	1281	1262	1565	1790	7733
Sex(0–5 year)							
Male	3493	6799	7552	5989	6520	6918	37271
Female	2113	4425	4896	4127	4528	4553	24642
Occupation(0–5 year)							
Scattered children	4217	7721	9856	6569	7742	9090	45195
Nursery children	1386	3494	2583	3538	3287	2370	16658
Other	3	9	9	9	19	11	60
Pathogen(0–5 year)							
CoxA16	42	241	92	214	57	111	757
EV71	222	315	87	82	429	15	1150
Other EV	254	341	258	250	249	462	1814

Of 61913 HFMD cases, 37271 were males and 24642 were females, with an average male-to-female sex ratio 1.51 (1.65:1 in 2013,1.53:1 in 2014,1.54:1 in 2015,1.44:1 in 2016, 1.44:1 in 2017, and 1.52:1 in 2018 respectively). Table 1 showed that the majority (99.90%) of HFMD cases were preschoolers including 73.00% scattered children and 26.91% nursery children. 6072 specimens were collected from 61913 HFMD cases with the positive rate 61.28%. Among these positive specimens, other EV was the major pathogens accounting for 48.75%, followed by EV71 and CoxA16 accounting for 30.91% and 20.34% respectively (Table 1).

### Seasonal pattern

**Fig 1:**
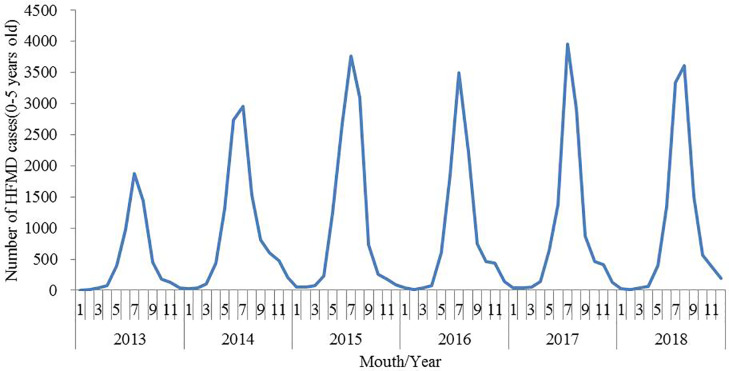
Monthly number of HFMD cases in Qingdao City, 2013–2018.

Fig 1 illustrated that the occurrence of HFMD in Qingdao City during 2013–2018 presented significant seasonality. The incidence of HFMD began to rise in May, and reached the peak in July each year. The cases reported during the high-incidence period (from May to October) accounted for 92.74% of the total number of cases.

### Geographic distribution and spatial autocorrelation of HFMD cases

**Fig 2:**
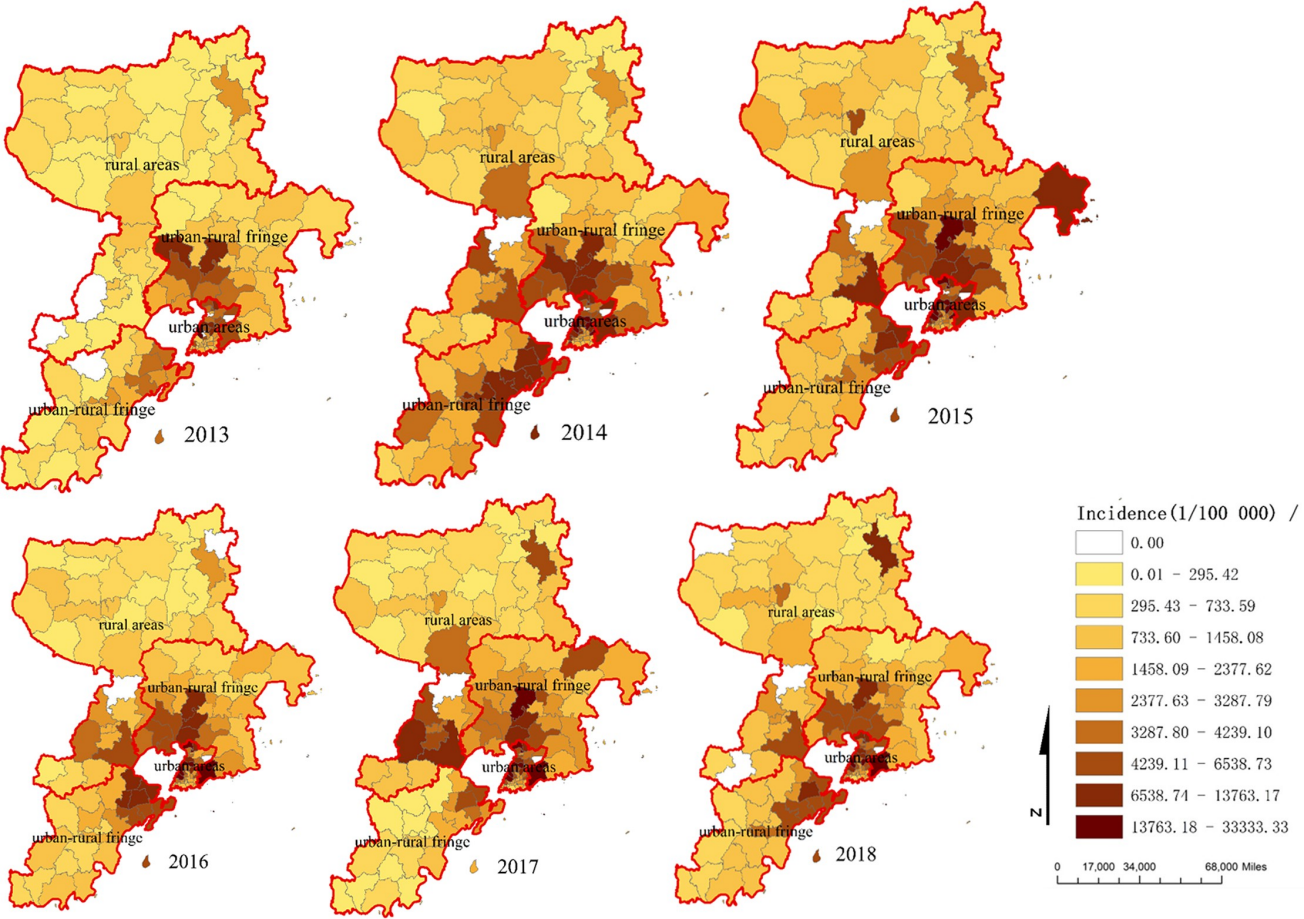
The incidence rate of HFMD among children aged 0–5 year old at street/township level in Qingdao City, 2013–2018.

The geographic distribution of HFMD in children 0–5 year old at town level was shown in Fig 2. It clearly indicated that the distribution of HFMD was heterogeneous at town level. There were 47 towns of which the average incidence rate higher than that of the whole city. The locations of high-incidence town mainly concentrated on densely populated areas which included urban and urban-rural fringe areas with the average incidence rates for these areas 5409.60 per 100000 inhabitants and 3300.27 per 100000 inhabitants respectively. The rural areas had lower incidence rate with the average incidence rate 1154.54 per 100000 inhabitants. The difference of incidence in the three regions was statistically significant though statistical analysis (χ^2^ = 12427.40, P<0.05). Table 2 showed the results of the spatial autocorrelation test. The results indicated that the distribution of HFMD was nonrandom with the global Moran′*I* from 0.19 to 0.31 between 2013 and 2018.

**Table 2:** The results of the spatial autocorrelation test on HFMD cases in Qingdao Province, 2013–2018.

Year	Moran′s I	Z Score	P-value
2013	0.2574	8.9579	<0.001
2014	0.3135	10.2977	<0.001
2015	0.2764	9.1146	<0.001
2016	0.1851	6.3516	<0.001
2017	0.2659	8.7284	<0.001
2018	0.2510	8.4413	<0.001

### Purely spatial analysis

**Fig 3:**
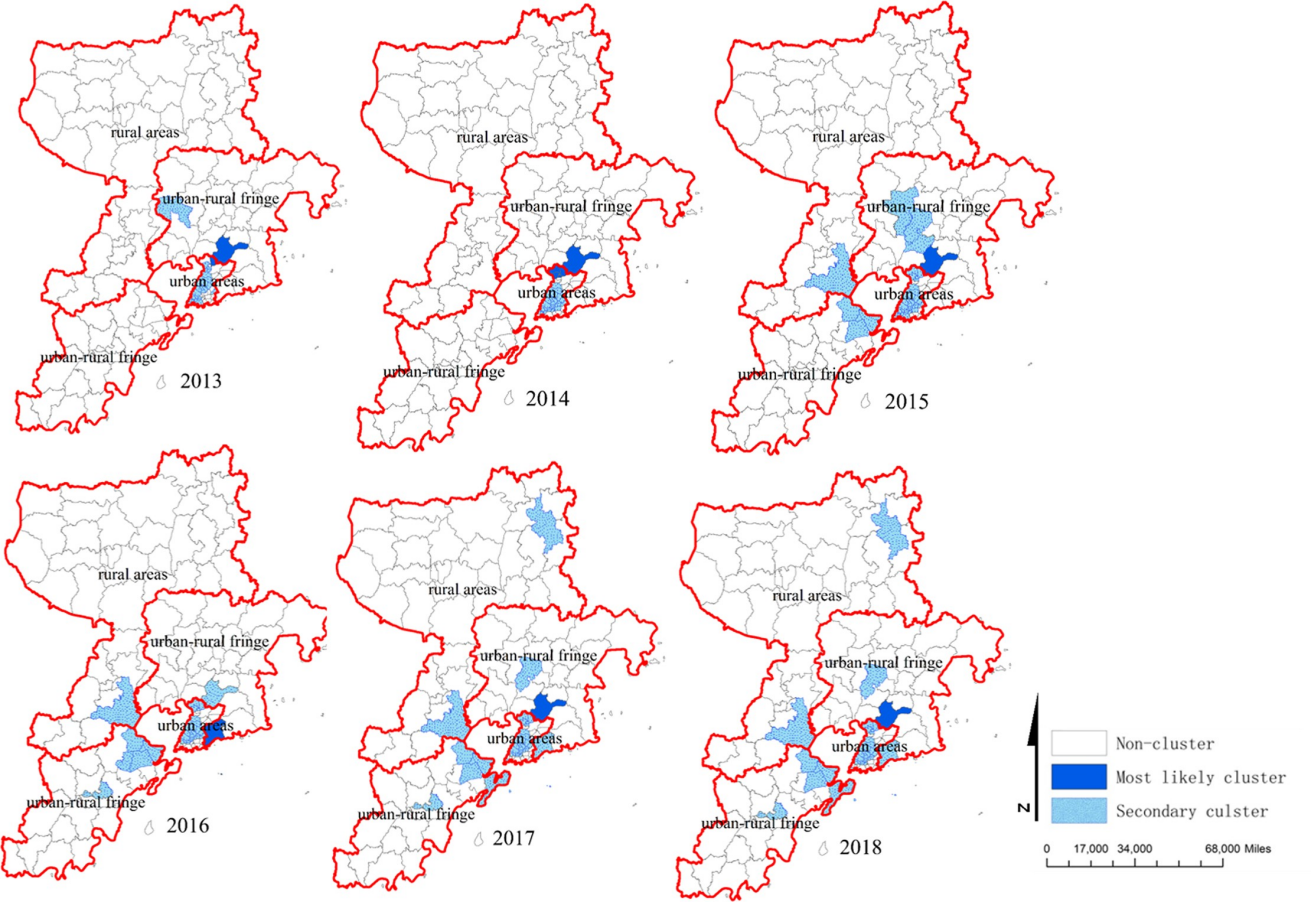
Spatial clusters of HFMD in Qingdao City, 2013–2018.

Using purely spatial scan statistic based on discrete Poisson model, the clusters which the sizes and locations varied each year were detected from 2013 to 2018. The most likely clusters were in Xiazhuang Street (2013, 2015, 2017, 2018), Xiangtan Street (2013, 2017), Xingrong Road, Jiaxing Road, Hailun Road(2014), Zhonghan Road (2016). The indexes of most likely clusters and the distribution of hotspots were showed in Table 3 and Fig 3.

**Table 3:** The most likely high risk clusters of HFMD cases detected using the purely spatial analysis.

Years	Clusters areas(n)	Radius(km)	Observed cases	Expected cases	Relative Risk	P-value
2013	2	7.19	258	77.46	3.45	<0.001
2014	24	6.98	1961	1156.05	1.86	<0.001
2015	1	0	381	72.77	5.38	<0.001
2016	1	0	444	119.24	3.86	<0.001
2017	2	7.19	570	149.79	3.97	<0.001
2018	1	0	363	61.66	6.06	<0.001

### Spatial-temporal clusters analysis

**Fig 4:**
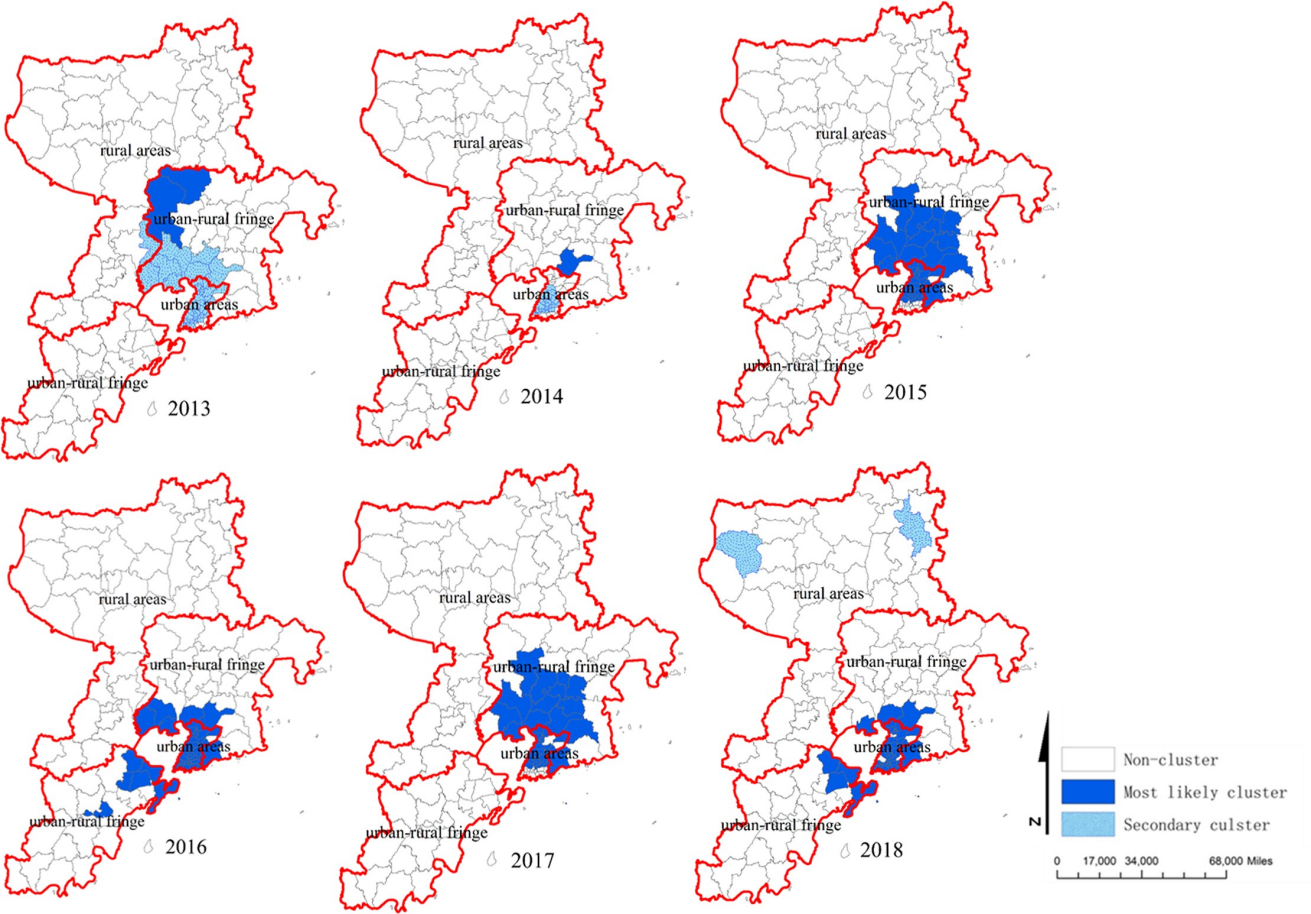
The spatial-temporal clusters of HFMD in Qingdao City, 2013–2018.

The spatial-temporal clusters were analyzed by space-time scan statistics method. 6 most likely cluster and 3 secondary clusters were found during the study period (Table 4). The most likely clusters in 2013 were located in Shibei, Licang, Shinan, Chengyang, Jimo District including 47 towns. The cluster time was from 1 Jun to 30 Sep 2013 and the radius was 18.97km with the RR 1.61. In 2014, the most likely clusters were located in Shibei, Shinan District including 24 towns. The cluster time was from 1 May to 31 Oct and the radius was 6.98km with the RR 1.88. The most likely clusters in 2015 and 2017 were Shibei, Licang, Chengyang District including 40 and 39 towns. The cluster times were all from 1 Jun to 30 Aug and the radius were all 19.66km with the RR were 1.87, 2.09. Similarly, in 2016 and 2018, the most likely clusters were in Shibei, Shinan, and Licang District including 53 and 49 towns. The cluster times were also from 1 Jun to 30 Aug and the radius were 25.03 and 22.92km with the RR were 1.82, 1.92. All the spatial-temporal clusters analysis of HFMD were shown in Table 4 and Fig 4.

**Table 4:** Spatial-temporal clusters analysis of HFMD in Qingdao City, 2013–2018.

Year	Cluster type	Cluster time	Radius(km)/towns(n)	Observed cases	Expected cases	RR	LLR	P-value
2013	Most-likely	Jun 1-Sep 30	18.97(47)	2478	1874.12	1.61	145.24	<0.001
	Secondary	May 1-Aug 31	12.53(4)	171	103.73	1.67	18.65	<0.001
2014	Most-likely	May 1-Oct 31	6.98(24)	1700	978.79	1.88	246.10	<0.001
	Secondary	May 1-Oct 31	0.00(1)	236	45.15	5.33	201.25	<0.001
2015	Most-likely	Jun 1-Aug 31	19.66(40)	4973	3323.46	1.87	529.81	<0.001
2016	Most-likely	Jun 1-Aug 31	25.03(53)	4222	2913.58	1.82	399.85	<0.001
2017	Most-likely	Jul 1- Aug 31	19.66(39)	3853	2285.85	2.09	607.18	<0.001
2018	Most-likely	Jul 1- Aug 31	22.92(49)	3844	2425.37	1.92	482.61	<0.001
	Secondary	Jun 1-Sep 30	10.41(2)	95	46.00	2.07	20.01	<0.001

## Discussion

In our study, we described the epidemiology of HFMD in Qingdao City from 2013 to 2018. We found that the incidence of HFMD presented an increasing trend and had been at a high level between 2015 and 2018, which was much higher than that of Shandong Province (93.70 per 100000) [24]. The result indicated that great pressures were still to be dealt with the prevention and control of HFMD in Qingdao City.

We observed that the children under 5 years old accounted for 88.90% of all cases, comparing well with other studies [25–28]. This could attribute to differences in antibodies level in different age groups. A seroepidemiological studies had shown that seropositivity to EV71 decreased rapidly from 1 month to 2 years old, increased gradually from 2 to 5 years old, and reached a stable state after 5 years old [29]. Another study in Zhejiang Province also found that the EV71 seroprevanlence in 6–10 years group and 11–20 years group (54.60% and 61.80% respectively) were significantly higher than that in 0–5 years group (29.10%) [30]. In addition, we found that scattered children (73.00%) accounted for a much larger proportion than nursery children (26.91%). Similarly, Liu et al showed that about half of HFMD cases in Nanchang, China, were scattered children [31]. And a number of studies also noted that a higher percentage of HFMD cases occurred among children who did not attend a nursery or preschool [32, 33]. One possible reason was that some prevention and control measures, such as hand-washing intervention, morning check, case isolation system, and school closure, were implemented in institutional settings in recent years. Previous studies of HFMD in other regions had shown that males were more susceptible to enterovirus than females, and the same was true of our findings [34–36]. This could be attributed to the fact that males were more active and more contacted favoring the spread of HFMD.

Although EV71 and CA16 were the most common etiological pathogens in many HFMD cases around the world [37, 38], our study showed that other EV was the major pathogens of HFMD in Qingdao during 2013–2018 except 2017. Notably, some other studies showed that other EV, such as CA6 and CA10, was going to become the important causative agents for HFMD in China, Finland, France, and Japan [39–41]. This suggested that other EV should be further classified to prevent HFMD cases associated with other new enteroviruses.

As in other reports [36, 37], the study showed that HFMD presented distinctly seasonality. The single seasonal peaks could be found between May and October. Some studies found that there were two kinds of peak patterns of HFMD epidemic in China: one peak in summer in northern China and two peaks in spring and autumn in southern China [42, 43]. These differences might be partly attributed to climatic factors, such as temperature and humidity [44].

Our study also found that HFMD cases in Qingdao City were mainly concentrated in urban and urban-rural fringe areas. Liao J et al [45] also had reported that the incidence of HFMD in urban areas with high population density was much higher than rural areas. By spatial autocorrelation analysis, the value of global Moran′*I* was from 0.19 to 0.31 between 2013 and 2018, indicating that the spatial distribution was not random at town level. This result was similar to the spatial autocorrelation patterns in China at city spatial scale level [24,37,45]. In order to better explore the spatial epidemic trend of HFMD, further spatial autocorrelation analysis should be done at village or community level.

Using purely spatial scan statistic based on discrete Poisson model, we found that the most likely clusters were in Xiazhuang Street (2013, 2015, 2017, 2018), Xiangtan Street (2013, 2017), Xingrong Road, Jiaxing Road, Hailun Road (2014), and Zhonghan Road (2016). These areas were respectively belonged to urban and urban-rural fringe areas with highly developed economy, high population density and mobility. The space-time scanning results found that the clusters almost occurred in May to October, which was basically the same as the seasonal peaks of HFMD in Qingdao City. Furthermore, the most likely cluster was located in urban and urban-rural fringe areas in Qingdao City during the whole study period, consistent with previous studies [45]. The reason for the urban areas as most likely cluster areas might be related with high-density populations, high-level medical diagnosis and cases report timely. It was worth noting that there were many “villages within cities” in urban-rural fringe areas with large number of migrant workers, backward municipal facilities, weak health awareness and poor health environment in China. Some studies indicated that many migrant workers lived with their children, most of whom were under 5 years old and not at school, which leaded to high incidence of HFMD in urban-rural fringe areas [38]. Moreover, the space-time scanning results indicated that the tendency of HFMD in Qingdao City spreaded from urban area to rural area. Therefore, this result reminded us that prevention and control measures should be implemented not only in urban area, but also in rural area which should be particularly strengthened.

Owning to adequately utilize the time and spatial information of data, the spatial-temporal clusters analysis could simultaneously locate the time and space of high incidence disease, and identify the high-risk time and areas of disease. To explore the dynamic characteristics of disease in temporal and spatial dimensions, the Scan software was better than the traditional descriptive mathematical statistical methods. Therefore, this method which was widely used to detect clusters of infectious disease could facilitate to adopt preventive and control measures in key areas.

Our study was the first time to analyze the spatial-temporal clusters of HFMD at town level in Qingdao City. In this study, we obtained more accurate high-incidence areas of HFMD, which could help to carry out prevention and control of HFMD purposefully and provide certain guiding significance on saving social resource. Despite insights gained from our study, there were still several limitations. First, we were only able to include HFMD cases reported to surveillance system. As a self-limiting illness, some patients may not attend hospital, which were not included in the surveillance system. Second, the dominant enteroviruses (other EV) were not further classified to identify new enteroviruses.

In summary, our study illuminated the spatial-temporal epidemiological characteristics of HFMD at town scale level from 2013 to 2018 in Qingdao City. The results revealed that urban and urban-rural fringe areas were the major locations of the clusters with other EV as the dominant pathogen. With the help of the finding, the prevention and control of HFMD in Qingdao City should be focus on the high-risk periods and locations which had important public health significance for the allocation of public health resources.

## Supporting information

S1 Data (XLS)
